# Trends in emigration of Polish health professionals. Where are we heading?

**DOI:** 10.1093/eurpub/ckac131.374

**Published:** 2022-10-25

**Authors:** A Domagała, M Kautsch, A Kulbat, K Parzonka

**Affiliations:** Jagiellonian University Medical College, Krakow, Poland

## Abstract

**Background:**

Migration of health professionals is one of the key challenges for many healthcare systems. In Poland this phenomenon is still under-researched. The aim of our study was to explore the estimated trends and directions of emigration among Polish health professionals.

**Methods:**

The research was based on the data analysis of specifying the number of people who applied for the certification of their professional qualifications (the right to practice a profession) at five national registers maintained by chambers of health professionals: 1) doctors and dentists, 2) nurses and midwives, 3) physiotherapists; 4) pharmacists and 5) laboratory diagnosticians. The gathered data reported information which allows for an approximate determination of how many professionals are considering a decision to migrate from Poland. Additionally exploration of data from the European Commission Regulated Profession Database in the EU Single Market was performed.

**Results:**

About 7-9% of Polish doctors and nurses have applied for certificates, which confirm their right to practice their profession in other European countries. The number of such certificates applied for by physiotherapists is also worrying. Emigration among pharmacists and laboratory diagnosticians is rather marginal. The biggest number of certificates, was issued to health professionals in the years 2004-2007, right after Poland joined the EU. In the period 2008-2015 the trend was not constant and it was related to the healthcare reform and changes in health professionals’ remuneration. Since 2016 the number of health workers applying for the certificates is by to a certain extent falling. The main destination of Polish emigrants were and are: United Kingdom, Germany, Sweden, Spain, Ireland, and EFTA countries (Norway, Switzerland).

**Conclusions:**

Implementation of a mechanism for monitoring emigration is necessary, but systemic improvement of working conditions in Polish healthcare system is also needed.

**Key messages:**

• The emigration, especially of young generations of medical staff, causes significant problems for the Polish healthcare system.

• More in-depth research on migration of health professionals is necessary.

